# Appropriateness, Reasons and Independent Predictors of Consultations in the Emergency Department (ED) of a Dutch Tertiary Care Center: A Prospective Cohort Study

**DOI:** 10.1371/journal.pone.0149079

**Published:** 2016-02-19

**Authors:** Daniël van der Veen, Christian Heringhaus, Bas de Groot

**Affiliations:** Emergency Department, Leiden University Medical Center, Leiden, the Netherlands; Mayo Clinic, UNITED STATES

## Abstract

**Objective:**

Consultations occur frequently in the emergency department (ED) of tertiary care centres and pose a threat for patient safety as they contribute to ED lengths of stay (LOS) and overcrowding. The aim of this study was to investigate reasons and appropriateness of consultations, and the relative impact of specialty and patient characteristics on the probability of a consultation, because this could help to improve efficiency of ED patient care.

**Methods:**

This prospective cohort study included ED patients presenting to a Dutch tertiary care centre in a setting where ED physicians mostly treat self-referred and undifferentiated patients and other specialists treat referred patients. Consultations were defined as appropriate if the reason of consultation corresponded with the final advice, conclusion or policy of the consulted specialty. Multivariable logistic regression analysis was used to assess the relative contribution of specialty and patient characteristics on consultation.

**Results:**

In the 344 (24% (95% CI 22 to 26%)) of the 1434 inclusions another specialty was consulted, resulting in a 55% increase of ED LOS. ED physicians more often consulted another specialty with a corrected odds ratio (OR) of 5.6 (4.0 to 7.8), mostly because consultations were mandatory in case of hospitalization or outpatient follow-up. Limited expertise of ED physicians was the reason for consultation in 7% (5 to 9%). The appropriateness of consultations was 84% (81 to 88%), similar between ED physicians and other specialists (P = 0.949). The patient characteristics age, comorbidity, and triage category and complaint predicted consultation.

**Conclusion:**

In a Dutch tertiary care centre another specialty was consulted in 24% of the patients, mostly for an appropriate reason, and rarely because of lack of expertise. The impact of consultations on ED LOS could be reduced if mandatory consultations are abolished and predictors of a consultation are used to facilitate timely consultation.

## Introduction

### Background

Worldwide, consultations are commonly requested in the emergency department (ED) [1–3] and pose a threat for patient safety as they contribute to longer ED length of stay (LOS) and consequently ED overcrowding [4–7]. Especially in tertiary care centres this is worrisome because a recently published systematic review indicated that the number of ED consultations in these centres is approximately twice as high compared to (sub)urban hospitals [1]. This could be explained by the presence of (sub)specialists in these tertiary care centres because, on the one hand, limited capabilities of (sub)specialists to make decisions outside their expertise forms a risk for an excessive number of consultations in the ED, leading to inefficient patient care, dissatisfaction among patients and staff and decreased cost effectiveness [4–5, 8]. On the other hand, if patients are properly referred by general practitioner (GP) to a (sub)specialist this might limit the number of consultations because ED physicians are not needed as an extra doctors in the chain of care.

The presence of both ED physicians and other specialists in the Dutch ED setting provides a unique possibility to study the effect of specialty (ED physician versus other specialists) on the consultation process in tertiary care centers, which could facilitate the optimization of the consultation process. In most Dutch tertiary care centres, as well as in the study centre, ED physicians are responsible for self-referred patients, patients who are referred to the ED physician and all shock-room presentations, including trauma care and cardiac arrests, in which the ED physicians are the team leader. In addition, they perform a primary assessment in all patients with the Manchester triage category (MTS) orange or red [9]. Finally, ED physicians provide procedural sedation and analgesia in the ED and are responsible for ED logistics and overall safety.

Residents of the other specialties take care of patients who have been specifically referred to them by either the general practitioner (GP) or other specialists with often typical tertiary care pathology, such as complications after transplantation and hemato-oncologic disorders.

### Importance

The negative effect of consultations on ED LOS and overcrowding are generally accepted [4–6, 10–13]. Insight in the reasons and appropriateness of consultations is necessary to reveal which consultations can be abolished, improving efficiency of patient care by reducing ED LOS and overcrowding. In addition, assessment of the relative impact of specialty (ED physician vs other specialist) and patient characteristics, on the probability of a consultation, will provide insight in the efficiency of how patient care is divided over ED physicians and (sub)specialists. For example, if ED physicians always have to consult another specialty due to lack of expertise, they are a redundant doctor in the chain of care. In this case, efficiency would be better if patients would have been directly referred to other (sub)specialists. A prediction model in which specialty and patient characteristics are incorporated has the additional benefit to be used to facilitate consultation in an early stage after ED presentation, also contributing to reduction of ED LOS and overcrowding.

### Objectives

The aim of this study is therefore two-fold: Firstly, to investigate the number, reasons and appropriateness of ED consultations. Secondly, to assess the relative contribution of specialty (ED physician vs other specialty) compared to patient characteristics on the probability of a consultation.

## Methods

### Study design and setting

This was a prospective observational cohort study in the ED of the Leiden University Medical Center (LUMC), a Dutch tertiary care centre with ~30.000 ED visits annually, between December 16 2014 and February 11 2015.

In the LUMC, the ED is staffed 24/7 by ED physicians, who supervise an emergency medicine resident and/or a physician assistant, and by residents of other specialties.

The study was approved by the medical ethics committee of the LUMC, who waived the need for individual informed consent because of the purely observational character of the study.

### Selection of participants

All consecutive patients presenting to the ED between 10 a.m. and 10 p.m. were included during 30 randomly chosen days, including weekends, over a 6 weeks period. Retrospective data indicated that approximately 70% of all patients per 24 hours visit the ED in the aforementioned time period. Importantly, a previous study showed that this way of sampling does not introduce selection bias [14].

### Data collection

Demographics, comorbidities, type of arrival (by ambulance or ambulatory), referral status, MTS category [9], presenting complaint according to the MTS, time of departure and final disposition were prospectively recorded by an observer in a standard data collection form in SPSS (SPSS, V.20.0, IBM, New York, USA). The treating physician was recorded as ED physician or other specialist. Consultation status was recorded as no consultation, consultation or multidisciplinary resuscitation. The ED length of stay was calculated by subtracting the ED registration time, as registered in the digital hospital information system (Chipsoft, Amsterdam), from the time that the patient physically left the ED.

Comorbidities were quantified according to the Charlson comorbidity index (CCI) [15–16] and categorized as “low” if the index was below four or “high” if the index was four or higher.

In case of one or multiple consultations the number of consultation(s), consultation reason(s), consulted specialty(ies) and requesting specialist(s) were registered by the observer. The consulting physician was asked to clarify the consultation reason before the consulted physician reported his or hers conclusion back to the consulting physician. If a consultation took place after 10 p.m. this consultation was considered as a missing.

Finally, in all patients it was assessed if they had an unanticipated revisit within 48 hours with a complaint related to the primary visit [7].

All patient records were de-identified and analysed anonymously.

### Primary outcome measures

The primary outcome of the present study was appropriate consultation which is defined below.

#### Definitions

A consultation was defined as a situation in which one physician requested the professional opinion of another specialty for one or more of the following reasons. Reasons for mandatory consultation:

- ‘presumed need for admission to ward’
- ‘presumed need for intensive care unit (ICU) admission’
- ‘presumed need for outpatient follow-up’
- ‘pre-existing agreement’ (e.g. in case of head trauma with a CT scan indication it was obliged for the ED physician to consult a neurologist)

or

- ‘procedure for which a specific expertise is needed’, in which the requested procedure must be performed outside the ED (e.g. operation).

Reasons for consultations which are not mandatory:

- ‘exclusion of a specific diagnosis.’ For example, in patients with chest pain, an ED physician consults the cardiologist with the request to rule out acute coronary syndrome / unstable angina pectoris by admitting the patient and follow cardiac enzymes and perform an exercise test or imaging.
- ‘procedure for which a specific expertise is needed’, in which the requested procedure is performed in the ED (e.g. fiber endoscopy).

or

- ‘other’

Consultations were performed by residents supervised by a staff member. Residents and physician assistants in emergency medicine were directly supervised by the ED physician, who was physically present 24/7 in the ED. In contrast, residents of other specialties were mostly supervised over the telephone, as staff members were mostly not physically present in the ED.

Radiology requests for specific diagnostic procedures, for example the request of an ultrasound, were not considered as consultations [2].

A consultation was defined as appropriate if the consult reason corresponded with the final advice, conclusion or policy of the consulted specialty.

Consultations were stratified in ‘mandatory’ and ‘none mandatory’ consultations for several reasons:

1. In contrast to other specialties, ED physicians had to consult another specialty if a hospitalization (including for operation) or outpatient follow-up was needed. These mandatory consultations contribute to relatively high numbers consultations by ED physicians, and to lead to an overestimation of the impact of specialty on consultation.
2. By definition, aforementioned mandatory consultations result in hospitalisation. The impact of patient characteristics associated with hospitalisation, such as high acuity presentations, will be overestimated. Because ED physicians almost exclusively treat these high acuity patients, the impact of specialty (ED physicians) on the probability of a consultation will be overestimated.
3. The appropriateness of a consultation between mandatory and none mandatory consultation may differ, potentially affecting the relative contribution of specialty compared to patient characteristics on consultation.

### Data analysis

Continuous data were presented as mean (standard deviation: SD) if normally distributed, and as median (interquartile range: IQR) if skewed. Categorical data were presented as number (%). Continuous data were compared using student t-tests or Mann-Whitney *U*-tests, as appropriate. Descriptive categorical data were analyzed using chi-square tests or Fisher’s exact tests, as appropriate.

Multivariable binary logistic regression analysis was used to identify independent predictors of consultation. The variables age, triage category and arrival by ambulance were forced into the model because a previous study had found that these were potential predictors of consultation [2]. Furthermore, the variable ‘treating physician’ was forced into model because we were specifically interested in the relative impact of specialty (ED physician versus other specialists) on the probability of a consultation. To identify additional potential predictors, forward entry of variables with a P-value <0.2 in the univariate analysis was used.

To prevent overfitting, the rule of thumb was taken into account that at least 10 events were needed per predictor variable used in the model. The prognostic and discriminative performance of the model was quantified by c-statistics with an area under the curve (AUC) analysis and the Hosmer-Lemeshow test, respectively. The odds ratios (OR) with 95% confidence intervals (95% CI) were reported. P-values below 0.05 were considered to be significant.

All data were analyzed with SPSS (V.20.0, IBM, New York, USA).

#### Sensitivity analysis

To assess collinearity, variance inflation factors (VIFs) were assessed in advance of the multivariable analysis. A VIF of 3 or higher was considered to indicate collinearity. To confirm the absence collinearity between the variable ‘arrival by ambulance’ and ‘triage category’, two additional multivariable analyses were performed. The first analysis included the variable ‘arrival by ambulance’, while the variable ‘triage category’ was excluded, while the second analysis included the variable ‘triage category’ and excluded the variable ‘arrival by ambulance’. A similar analysis was performed to confirm the absence collinearity between the variable ‘treating physician’ and ‘triage category’.

## Results

### Patient characteristics

**Fig 1:**
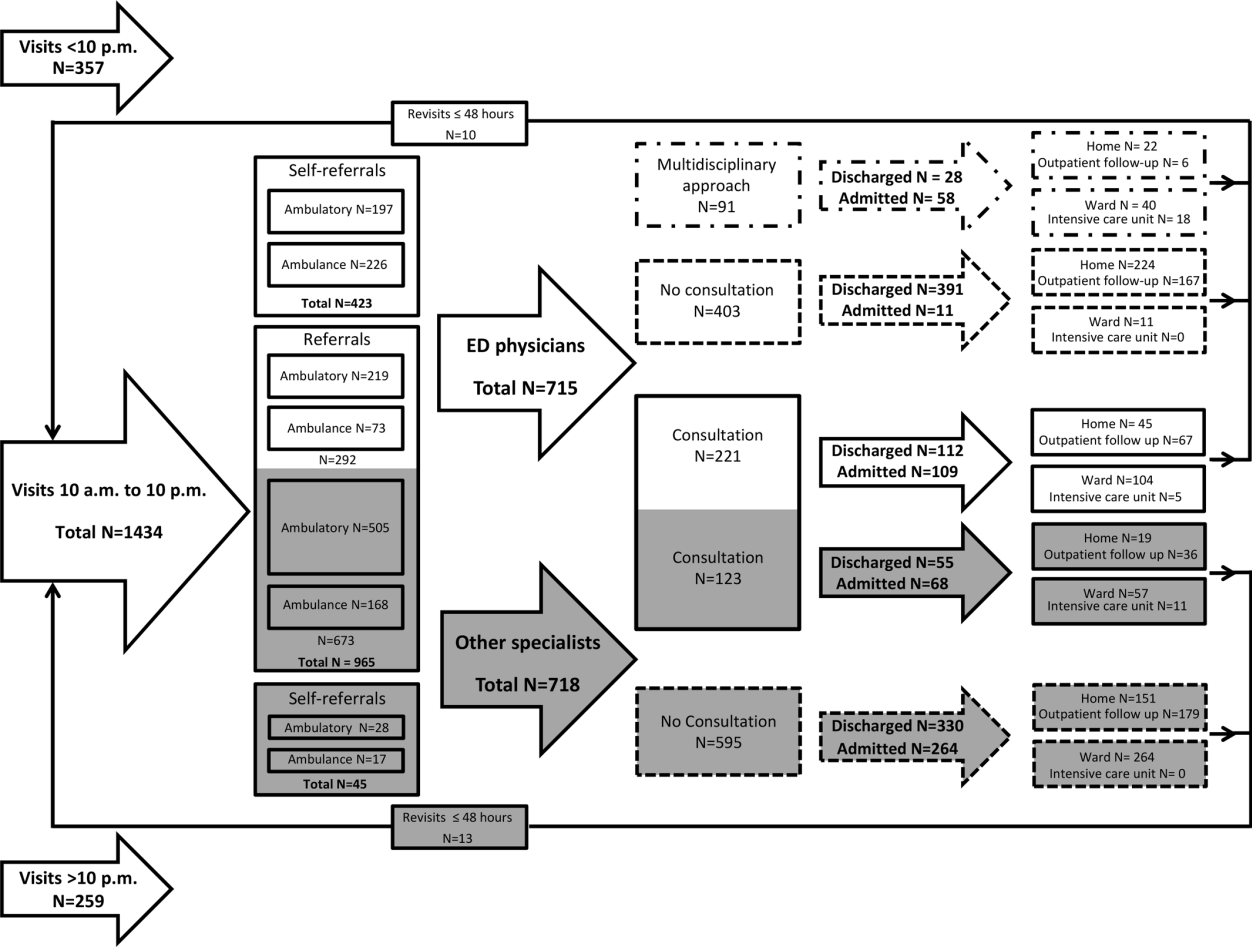
Input, throughput and output flow diagram of included patients. Patients treated by the ED physician are depicted in white, and those treated by the other specialties in grey. The boxes with dashed lines present patients that received no consultation or multidisciplinary approach. When the lines are closed, patients received one or multiple consultations. One patient was admitted to the ward without being treated by any physician. In 1 patients the final disposition was unknown. Six patients had another final disposition as depicted, of whom 5 had died on the ED and 1 returned to the psychiatric hospital were the patient was admitted before arrival at the ED.

Of the 2050 patients visiting the ED during the study period, 1434 (70%) visited during the inclusion hours from 10 a.m. until 10 p.m. An overview of the patient flow is provided in Fig 1. Patient characteristics are shown in Table 1 and S1 Table. In S2 Table, in which the total population was stratified according to treating physician, it is shown that ED physicians and other specialties treated different patient populations. ED physicians treated 715 (50% (47 to 52%)) of the 1434 patients, of which 91 (13% (10 to 15%)) received multidisciplinary resuscitation in the shock-room. Patients treated by the ED physician more often were self-referred (59 versus 6%) and more often arrived by ambulance (42 versus 26%). Furthermore, they were more often triaged ‘red’ (5 versus 0%) or ‘green’ (30 versus 18%) and had a lower CCI (0.64 versus 1.42). Twenty-three (2% (1 to 4%) patients had an unanticipated revisit within 48 hours, with no difference as to whether they had received a consultation or not (P = 0.228). Ten of these 23 patients were treated by the ED physicians and 13 by other specialists (P = 0.538).

**Table 1:** Patient characteristics.

	Total population	No Consultation	Consultation	p-value
**N** (%) *	1434	999 (70)	344 (24)	
**Demographics**				
Age, mean (SD)	48 (26)	44 (25)	55 (25)	<0.001
Sex (male, %)	749 (52)	514 (51)	186 (54)	0.412
Pediatrics (%)	239 (17)	189 (19)	42 (12)	0.004
**CCI**, mean (SD) **	1.03 (1.70)	0.93 (1.61)	1.31 (1.94)	0.001
Low (%)	1311 (91)	930 (93)	298 (87)	<0.001
High (%)	123 (9)	69 (7)	46 (13)	
**Referral status**				
Self-referral	469 (33)	259 (26)	142 (41)	<0.001
General practitioner	612 (43)	454 (45)	137 (40)	0.107
Specialist	353 (25)	286 (29)	65 (19)	<0.001
**Arrival by ambulance** [1]	484 (34)	221 (22)	174 (51)	<0.001
**Triage category** [4] ^#^				
Red	32 (2)	1 (0)	5 (1)	0.006
Orange	371 (26)	203 (20)	114 (33)	<0.001
Yellow	682 (48)	491 (49)	182 (53)	0.118
Green	337 (24)	294 (29)	42 (12)	<0.001
Blue	8 (1)	7 (1)	1 (0)	0.689
**Treating physician** [1]				
ED physician	715 (50)	403 (40)	221 (64)	<0.001
Internal medicine	221 (15)	180 (18)	41 (12)	0.005
Cardiology	117 (8)	86 (9)	31 (9)	0.945
Surgery	71 (5)	63 (6)	8 (2)	0.003
Neurology	78 (5)	56 (6)	22 (6)	0.500
Other specialties	231 (16)	210 (21)	21 (6)	<0.001
**Disposition** [1]				
Home	461 (32)	375 (38)	64 (19)	<0.001
Outpatient follow-up	455 (32)	346 (35)	103 (30)	0.109
Admission ward	477 (33)	276 (28)	161 (47)	<0.001
Admission ICU	34 (2)	0 (0)	16 (5)	<0.001
Other ^##^	6 (0)	1 (0)	0 (0)	0.557
**ED LOS** (minutes), median (IQR) [333]	146 (91 to 209)	131 (82 to187)	203 (152 to 273)	<0.001
**Revisits ≤ 48 hours**	23 (2)	19 (2)	3 (1)	0.228

Patient characteristics are presented for the total population, patients who received no consultations and patients who received one or multiple consultations. Continuous data are presented as mean (SD) or median (IQR) and categorical data as frequency (%). The number of missing cases are noted between square brackets for each variable. Revisits ≤ 48 hours includes only patients that revisited the ED unanticipated with a complaint related to the index visit.

* A total of 91 patients received multidisciplinary resuscitation in the shock-room. These patients were not counted in the columns ‘no consultations’ and ‘consultation, therefore the numbers in the columns do not always add up to the number in the ‘total’ column.

** Patients with a CCI of ≤3 were classified as low and those with a CCI ≥4 as high.

# The presented ‘triage category’ was according to the Manchester Triage System (MTS).

## Of the 6 patients with another final disposition, 5 patients died on the ED and 1 patients returned to the psychiatric hospital were the patient was admitted before arrival at the ED. Abbreviations: CCI, Charlson Comorbidity Index; ED, Emergency Department; ICU, Intensive Care Unit; LOS, Length of Stay.

### Frequency, reasons and appropriateness of consultations

Prospective assessment of frequency, reasons and appropriateness of consultations was necessary to investigate if there were consultations which could potentially be abolished, improving efficiency of ED patient care.

Another specialty was consulted in 344 (24% (22 to 26%)) of the 1434 ED patients. The number of consultations was highest in patients treated by the ED physicians (31 versus 17%). In 22 (3% (2 to 4%)) of the 715 patients treated by the ED physicians two or more other specialties were consulted, compared to 19 (3% (1 to 4%)) of the 718 patients treated by the other specialists (P = 0.888). This led to a total of 388 requested consultations. In 30 (8%, 5% to 10%) consultations, the reason of consultation was missing, as these consultation were requested after 10 p.m. The overall appropriateness of consultations was 85% (80 to 90%) for the ED physicians and 81% (74 to 88%) for the other specialists (P = 0.949).

#### Mandatory consultations

In Table 2 the reasons and the number of appropriate and inappropriate mandatory consultations of ED physicians and other specialties are shown to get insight in the number of consultations which may be unnecessary. S3 Table provides the patients characteristics of these 169 patients.

**Table 2:** Appropriateness of mandatory consultations.

	Total	ED physicians			Other specialists		
		Total	Appropriate	Inappropriate	Total	Appropriate	Inappropriate
**N** (%)	**180**	**129**	**114 (88)**	**15 (12)**	**51**	**41 (80)**	**10 (20)**
**Admission ard (%)**	**71 (39)**	**66**	**55 (83)**	**11 (17)**	**5**	**3 (60)**	**2 (40)**
Internal medicine		27	25	2	2	0	2
Cardiology		8	6	2	-	-	-
Surgery		14	14	0	-	-	-
Neurology		5	3	2	-	-	-
Other		12	7	5	3	3	0
**AdmissionICU (%)**	**19 (11)**	**3**	**3 (100)**	**0**	**16**	**8 (50)**	**8 (50)**
**Outpatient follow-up (%)**	**7 (4)**	**7**	**5 (71)**	**2 (29)**	**0**	**0**	**0**
**Pre-existing agreement (%)**	**63 (35)**	**34**	**34 (100)**	**0 (0)**	**29**	**29 (100)**	**0 (0)**
Cardioversion		2	2	0	17	17	0
First seizure		3	3	0	-	-	-
CT-cerebrum		10	10	0	-	-	-
Trauma		-	-	-	10	10	0
Other		19	19	0	2	2	0
**Specific procedure (%)**	**20 (11)**	**19**	**17 (89)**	**2 (11)**	**1**	**1 (100)**	**0 (0)**
Operation		16	14	2	1	1	0
GI endoscopy		3	3	0	-	-	-

The number of appropriate and inappropriate mandatory consultations requested by ED physicians and by other specialists. Data are presented as frequency (%).The percentages in the appropriate and inappropriate columns presents the appropriateness of the consultations requested for that specific reason by that specific specialty. The percentages in the total consultation column presents the percentage of obliged consultation requested for that specific reason. Abbreviations: CT, Computed tomography; ED, Emergency department; ICU, Intensive care unit; GI endoscopy, Gastro-intestinal endoscopy.

In 169 (12% (10 to 13%)) of the 1434 patients a consultation of another specialty was mandatory. ED physicians more often requested mandatory consultations compared to other specialists (17 versus 6%). In 11 patients multiple mandatory consultation were requested. Thus, 180 (46% (41 to 51%)) of the 388 consultations were mandatory, in 86% for an appropriate reason.

The reasons ‘presumed need for admission to ward’, ‘presumed need for outpatient follow-up’ and ‘procedure for which a specific expertise is needed’ were the most common reasons for a mandatory consultation, and were mostly requested by ED physicians.

Consultations for ‘presumed need for ICU admission’ was adequate in 100% (3 of 3) of patients treated by the ED physician and in 50% (8 of 16) of the patients treated by the other specialists (P = 0.228).

#### None mandatory consultations

In 155 (11% (9 to 12%)) of 1434 patients the requested consultation was not mandatory. Patients characteristics of these 155 patients are shown in S3 Table. ED physicians and other specialist performed a similar number of none mandatory consultations (12 versus 9%, P = 0.047). In 7 patients multiple consultations for none mandatory reasons were requested. Sixteen patients received a mandatory and none mandatory consultation. This led to 178 (46% (41 to 51%)) of the 388 consultations being requested for a none mandatory reasons. The reasons and the number of appropriate and inappropriate none mandatory consultations is given in Table 3.

**Table 3:** Appropriateness of none mandatory consultations.

	Total	ED physicians			Other specialists		
		Total	Appropriate	Inappropriate	Total	Appropriate	Inappropriate
**N (%)**	**178**	**97**	**78 (80)**	**19 (20)**	**81**	**66 (81)**	**15 (19)**
**Specific procedure**	**44 (25)**	**31**	**30 (97)**	**1 (3)**	**13**	**11 (85)**	**2 (15)**
Read-out of ICD		9	9	0	-	-	-
Fiber endoscopy		3	3	0	-	-	-
Other		19	18	1	13	11	2
**Other**	**63 (35)**	**28**	**25 (89)**	**3 (11)**	**35**	**27 (77)**	**8 (23)**
**Exclusion specific diagnosis**	**71 (40)**	**38**	**23 (61)**	**15 (39)**	**33**	**28 (85)**	**5 (15)**
ACS		15	6	9	1	1	0
Other		23	17	6	32	27	5

The number of appropriate and inappropriate none mandatory consultations requested by ED physicians and by other specialists. Data are presented as frequency (%).The percentages in the appropriate and inappropriate columns presents the appropriateness of the consultations requested for that specific reason by that specific specialty. The percentages in the total consultation column presents the percentage of obliged consultation requested for that specific reason. Of the 9 patients with an inappropriate consultation none returned within 30 days with a major adverse cardiovascular event. Abbreviations: ACS, Acute coronary syndrome; ED, Emergency department; ICD, Implantable cardioverter defibrillator.

The appropriateness of none mandatory consultations was 80% (73 to 88%) for the ED physicians and 81% (73 to 90%) for the other specialists (P = 0.857). ED physicians only consulted another specialty more often for the none mandatory consultations ‘procedure for which a specific expertise is needed’ in which the procedure was performed in the ED (4 versus 2%, P = 0.016). This was appropriate in 30 (97%, 91 to 103%) of the 31 consultations.

### Impact of specialty and patient characteristics on the probability of a consultation

Assessment of the relative impact of specialty (ED physician vs other specialist) and patient characteristics, on the probability of a consultation, provides insight in the efficiency of how patient care is divided over ED physicians and (sub)specialists. A prediction model in which specialty and patient characteristics are incorporated can also be used to facilitate consultation in an early stage after ED presentation. In Table 4 it is shown that treating physician (OR 5.56 (3.99 to 7.76)) and triage category (OR 3.07 (1.91 to 4.95)) are the most important independent predictors. The Hosmer-Lemeshow goodness of fit test had a p-value of 0.229 and the AUC was 0.786 (0.757 to 0.814).

**Table 4:** Multivariable logistic regression analysis to predict consultation.

	Univariate analysis OR (95% CI)	Multivariable analysis Corrected OR(95% CI)	Multivariable analysis excluding mandatory consultations Corrected OR (95% CI)
**Age**	1.02 (1.01 to 1.02)	1.02 (1.01 to 1.02)	1.02 (1.01 to 1.03)
**CCI** *			
Low	Ref	Ref	Ref
High	2.08 (1.40 to 3.09)	2.12 (1.32 to 3.41)	2.19 (1.21 to 3.94)
**Triage category** **			
Green and blue	Ref	Ref	Ref
Yellow	2.60 (1.81 to 3.73)	1.82 (1.20 to 2.76)	1.76 (0.99 to 3.13)
Red and orange	4.08 (2.76 to 6.04)	3.07 (1.91 to 4.95)	2.79 (1.46 to 5.34)
**Treating physician**			
Other specialty	Ref	Ref	Ref
ED physician	2.65 (2.06 to 3.42)	5.56 (3.99 to 7.76)	4.62 (3.01 to 7.10)
**Arrival by ambulance**	3.60 (2.78 to 4.67)	2.21 (1.63 to 3.00)	1.77 (1.18 to 2.63)
**Triage complaint** **			
Other	Ref	Ref	Ref
Headache	1.30 (0.43 to 3.90)	2.71 (0.85 to 8.70)	4.13 (1.01 to 16.89)
Dyspnoea	0.95 (0.61 to 1.49)	0.95 (0.58 to 1.65)	1.09 (0.51 to 2.30)
Chest pain	0.97 (0.60 to 1.58)	0.49 (0.28 to 0.87)	1.20 (0.60 to 2.37)
Palpitations	4.06 (2.07 to 7.95)	5.56 (2.57 to 12.03)	1.80 (0.58 to 5.63)
Abdominal pain	0.98 (0.62 to 1.54)	1.13 (0.68 to 1.88)	1.66 (0.85 to 3.23)
Malaise	0.85 (0.58 to 1.24)	1.02 (0.65 to 1.59)	1.76 (0.99 to 3.13)
Traumatic injury	0.47 (0.32 to 0.70)	0.40 (0.25 to 0.63)	0.46 (0.24 to 0.89)
Syncope	1.73 (0.85 to 3.55)	0.61 (0.28 to 1.36)	0.96 (0.35 to 2.64)

Multivariable binary logistic regression analysis was performed with forward entry of potential variables with P<0.2 in the univariate analysis (Table 1) or forced entry of variables based on previous studies. Data are presented as odds ratio (OR (95% CI). The right column presents the odds ratios during the univariate analysis. The Hosmer-Lemeshows test had a p-value of 0.229 and the area under the curve was 0.786 (0.757 to 0.814). The multivariable analysis of the right column the mandatory consultations (‘presumed need for admission to ward’, ‘presumed need for outpatient follow-up’, ‘procedure for a which specific expertise is needed’ in which the expertise requested was operation or gastro-intestinal endoscopy, or ‘pre-existing agreement’, N = 1154) were excluded. The Hosmer-Lemeshow test had a p-value of 0.275 and the area under the curve was 0.769 (0.729 to 0.809).

* Patients with a CCI of ≤3 were classified as low and those with a CCI ≥4 as high.

** The presented ‘triage category’ and ‘triage complaint’ were according to the Manchester triage system (MTS). Abbreviations: CCI, Charlson Comorbidity Index; MTS, Manchester Triage System; Ref, reference category for the OR, odds ratio.

If mandatory consultations were excluded the corrected OR for consultation by the ED physician decreased to 4.62 (3.01 to 7.10), The Hosmer-Lemeshow goodness of fit test of this second model had a p-value of 0.275 and the AUC was 0.769 (0.729 to 0.809).

#### Sensitivity analysis

The multicollinearity analysis indicated that there was no collinearity between any of the potential predicting variables. S4 Table provides the variance inflation factors for the potential predicting variables. In addition, no significant change in the final model was seen when either the variable ‘arrival by ambulance’ or ‘triage category’ was removed. Therefore, both variables were included in the final analysis (data not shown). Furthermore, the removal of the variable ‘treating physician’ or ‘triage category’ showed no significant change in the final model (data not shown).

## Discussion

The main finding of this study was that in the ED of a Dutch tertiary care centre, ED physicians and other specialists consult another specialty in only 24% of the patients, mostly for an appropriate reason. The impact of consultations on ED LOS could be reduced if mandatory consultations are abolished and predictors of a consultation are used to facilitate timely consultation.

The ED consultation rate of 24% in the present study was lower than the previously reported consultation rates of ~40% [1–3]. In the Dutch health care system the GP has an important role as gatekeeper for many patients visiting the ED [17]. The observation that ‘limited expertise’ was the reason for consultation in only 7% of all patients treated by ED physicians and in 2% of patients treated by other specialists indicates that GPs refer patients adequately to either the ED physician or another (sub)specialist. We hypothesize that this explains the higher consultation efficiency in the Dutch health care system compared to other health care systems in which the role of the GP is smaller and primary care is often delivered in the ED.

To the best of our knowledge, the present study is the first to prospectively investigate the reasons and appropriateness of consultations, while insight in the reasons and appropriateness of consultations helps to understand if and how the number of consultations can be reduced. It was found that in most cases physicians appropriately consult another specialty. Correspondent to previous studies we found that the reason ‘presumed need for admission’ was the most common reason for consultation by ED physicians [1, 5]. This can be explained by the fact that, in the Dutch health care system, the ED physicians are obligated to consult another specialty if the patients need hospitalization. However, the necessity of mandatory consultations is questionable given the high level of appropriateness. Especially if the negative effect of consultations on the ED LOS and overcrowding, which is associated with a decrease in quality of care, patient safety and survival [10–13] is considered.

ED physicians requested a similar number of consultations for none mandatory reasons as other specialists. Only the number of none mandatory consultations by ED physicians for the reason ‘procedure for which a specific expertise is needed’ was slightly larger, which is explained by the fact that ED physicians treat more self-referred patients with undifferentiated complaints. In addition, patients who were referred to the ED physician were more likely to present with an undifferentiated complaint, while patients with a differentiated complaint were more likely to be referred to other specialties. Therefore “ED physician” was still an independent predictor of a consultation, even when the mandatory consultations were excluded.

The impact of consultations on ED LOS could be reduced if mandatory consultations by ED physicians for hospital admission and outpatient follow-up are abolished. The high level of appropriateness in combination with the low level of ‘lack of expertise’ and ‘unanticipated revisits’, indicates that this is feasible.

Facilitation of timely consultation, i.e. early after triage, would be another way to reduce the impact of consultation on ED LOS. Our prediction model for consultations would be valuable because it could reduce the delay of requesting a consultation. In a Canadian study by Wood *et al*., ED patients in whom another specialty was consulted were older, had higher acuity presentations and arrived more often by ambulance compared to patients receiving no consultation [2]. Similar patient characteristics were independent predictors of consultations in the present study, suggesting that these are universal among different health care systems.

### Limitations

Although this study has several strengths, like the prospective design, real-time assessment of appropriateness of consultations and the direct comparison of the effect of specialty (ED physician versus other specialist) on consultations, it has also several weaknesses.

Firstly, this study was performed in the ED of a tertiary care centre, limiting generalizability to urban hospitals. However, a recent published systematic review reported that number of consultations was highest in tertiary care centres [1]. This indicates that these centres form an important setting for studying ED consultations. Although the ED setting in which this study was performed, with ED physicians and other specialties both treating ED patients, might be unique to the Dutch health care system, it allowed direct comparison of the effect of specialty on consultations.

Secondly, patients were only included between 10 a.m. and 10 p.m., possibly introducing selection bias. However, pilot data (not shown), including all consecutive ED patients during 72 hours, indicated no differences between patients who visited the ED between 10 a.m. and 10 p.m. and patients visiting outside these hours. In addition, Valley *et al*. showed that by use of a similar inclusion method, differences between the included cohort and total cohort were small and clinically not relevant [14].

Finally, the relative small patient volume of our ED possibly contributes to the short ED LOS compared to larger tertiary care EDs. Therefore, this study might underestimate the absolute impact of consultation upon ED LOS. Furthermore, the impact might be underestimated by including only patients from 10 a.m. to 10 p.m. During night hours most hospital ancillary services, such as laboratory testing and radiology, are short staffed, delaying results required by the consultant for their final recommendations. Additional delay might be caused by less available supervision by staff member during night hours. However, even with the relatively small patient volume and inclusion hours, consultation caused a 55% increase in ED length of stay.

## Conclusion

In a Dutch tertiary care ED staffed by both ED physicians and other specialists, consultations rates are relatively low, and mostly requested for an appropriate reason. The impact of consultations on ED LOS could be reduced if mandatory consultations for hospital admission or outpatient follow-up is abolished. Future studies should investigate if the impact of consultations on ED LOS can be reduced by of our prediction model.

## Supporting Information

S1 Database The database including the data of the 1434 included patients. (XLSX)

S1 Table Additional patient characteristics. (DOCX)

S2 Table Patient characteristics stratified according to treating physician. (DOCX)

S3 Table Patient characteristics of patient with mandatory and none mandatory consultations. (DOCX)

S4 Table The Variance Inflation Factors of the independent predictors of consultation. (DOCX)
