# Supplementary material for: Appropriateness, Reasons and Independent Predictors of Consultations in the Emergency Department (ED) of a Dutch Tertiary Care Center: A Prospective Cohort Study
Source: PLoS One. 2016 Feb 19;11(2):e0149079. doi: 10.1371/journal.pone.0149079 (PMC4760948; doi:10.1371/journal.pone.0149079)
Supplement: S1 Table — (DOCX) [file pone.0149079.s002.docx]

**Supporting Information**

**Supporting Table 1.** **Additional patient characteristics.**

|  | | **Total population** | **No Consultation** | **Consultation** | **p-value** |
| --- | --- | --- | --- | --- | --- |
| **N** (%)^*^ | | 1434 | 999 (70) | 344 (24) |  |
| **Medical history** | |  |  |  |  |
|  | Myocardial | 248 (17) | 139 (14) | 91 (26) | <0.001 |
|  | Vascular | 322 (22) | 189 (19) | 103 (30) | <0.001 |
|  | Pulmonary | 144 (10) | 92 (9) | 44 (13) | 0.058 |
|  | Neurologic | 74 (5) | 37 (4) | 23 (7) | 0.021 |
|  | Endocrine | 156 (11) | 90 (9) | 50 (15) | 0.004 |
|  | Renal | 54 (4) | 36 (4) | 17 (5) | 0.272 |
|  | Liver | 12 (1) | 8 (1) | 4 (1) | 0.516 |
|  | Gastro-intestinal | 31 (2) | 20 (2) | 6 (2) | 0.765 |
|  | Malignancy | 277 (19) | 194 (19) | 67 (20) | 0.982 |
|  | Miscellaneous | 42 (3) | 32 (3) | 7 (2) | 0.354 |
| **Triage complaint** [3] ^#^ | |  |  |  |  |
|  | Headache | 16 (1) | 10 (1) | 5 (1) | 0.240 |
|  | Dyspnoea | 144 (10) | 101 (10) | 37 (11) | 0.865 |
|  | Chest pain | 114 (8) | 80 (8) | 30 (9) | 0.791 |
|  | Palpitation | 41 (3) | 16 (2) | 25 (7) | <0.001 |
|  | Abdominal pain | 135 (9) | 93 (9) | 35 (10) | 0.759 |
|  | Traumatic injury | 323 (23) | 255 (26) | 46 (17) | <0.001 |
|  | Syncope | 43 (3) | 21 (2) | 14 (4) | 0.078 |
|  | Malaise | 274 (19) | 184 (18) | 60 (17) | 0.635 |
|  | Other | 344 (24) | 239 (24) | 92 (27) | 0.447 |

The additional patient characteristics are presented for the total population, patients that received no consultations and patients that received one or multiple consultations. Continuous data are presented as mean (SD) or median (IQR) and categorical data as frequency (%).The number of missing cases are noted between square brackets for each variable. * A total of 91 (6%) patient received multidisciplinary resuscitation. ^#^ The presented ‘triage complaint’ was according to the MTS. Abbreviations: MTS, Manchester Triage System.
