# Supplementary material for: Appropriateness, Reasons and Independent Predictors of Consultations in the Emergency Department (ED) of a Dutch Tertiary Care Center: A Prospective Cohort Study
Source: PLoS One. 2016 Feb 19;11(2):e0149079. doi: 10.1371/journal.pone.0149079 (PMC4760948; doi:10.1371/journal.pone.0149079)
Supplement: S2 Table — (DOCX) [file pone.0149079.s003.docx]

**Supporting Information**

|  | | **Total population** | **ED physician** | **Other specialties** | **p-values** |
| --- | --- | --- | --- | --- | --- |
| **N** (%) ^*^ | | 1434 | 715 (50) | 718 (50) |  |
| **Demographics** | |  |  |  |  |
|  | Age, mean (SD) | 48 (26) | 45 (26) | 51 (25) | <.001 |
|  | Sex (male, %) | 749 (52) | 365 (51) | 383 (53) | 0.385 |
|  | Pediatrics (%) | 239 (17) | 137 (19) | 102 (14) | 0.012 |
| **CCI**, mean (SD) ^**^ | | 1.03 (1.70) | 0.64 (1.34) | 1.42 (1.92) | <.001 |
|  | Low (%) | 1311 (91) | 681 (95) | 630 (88) | <.001 |
|  | High (%) | 123 (9) | 34 (5) | 88 (12) |  |
| **Referral status** | |  |  |  |  |
|  | Self-referral | 469 (33) | 423 (59) | 45 (6) | <.001 |
|  | General practitioner | 612 (43) | 180 (25) | 432 (60) | <.001 |
|  | Specialist | 353 (25) | 112 (16) | 241 (33) | <.001 |
| **Arrival by ambulance** [1] | | 484 (34) | 299 (42) | 185 (26) | <.001 |
| **Triage category** [4] ^#^ | |  |  |  |  |
|  | Red | 32 (2) | 31 (5) | 1 (0) | <.001 |
|  | Orange | 371 (26) | 180 (25) | 191 (27) | 0.538 |
|  | Yellow | 682 (48) | 286 (40) | 395 (55) | <.001 |
|  | Green | 337 (24) | 208 (30) | 129 (18) | <.001 |
|  | Blue | 8 (1) | 7 (1) | 1 (0) | 0.038 |
| **Triage complaint** [3] ^#^ | |  |  |  |  |
|  | Headache | 16 (1) | 2 (0) | 14 (2) | 0.004 |
|  | Dyspnoea | 144 (10) | 42 (6) | 102 (14) | <.001 |
|  | Chest pain | 114 (8) | 48 (7) | 66 (9) | 0.097 |
|  | Palpitation | 41 (3) | 10 (1) | 31 (4) | 0.001 |
|  | Abdominal pain | 135 (9) | 64 (9) | 71 (10) | 0.544 |
|  | Small traumatic injury | 323 (23) | 271 (38) | 52 (7) | <.001 |
|  | Syncope | 43 (3) | 34 (5) | 9 (1) | <.001 |
|  | Malaise | 274 (19) | 78 (11) | 196 (27) | <.001 |
|  | Other | 344 (24) | 166 (23) | 177 (25) | 0.524 |
| **Multidisciplinary resuscitation** | | 91 (6) | 91 (13) | 0 (0) | <.001 |
| **Consultations** | | 344 | 221 (31) | 123 (17) | <.001 |
| **ED LOS** (minutes), median (IQR) [333] | | 146 (91 to 209) | 117 (69 to 180) | 170 (124 to 228) | <.001 |
| **Revisits ≤ 48 hours** | | 42 (3) | 10 (1) | 13 (2) | 0.538 |

**Supporting Table 2. Patient characteristics stratified according to treating physician.**

Patient characteristics are presented for the total population, patients treated by the ED physicians and patients treated by the other specialists. Continuous data are presented as mean (SD) or median (IQR) and categorical data as frequency (%). The number of missing cases are noted between square brackets for each variable. Revisits ≤ 48 hours includes only patients that revisited the ED unanticipated with a complaint related to the index visit. * One patients was directly admitted to the ward without being treated by any physician. ** Patients with a CCI of ≤3 were classified as low and those with a CCI ≥4 as high. ^#^ The presented ‘triage category’ and ‘triage complaint’ were according to the MTS. Abbreviations: CCI, Charlson Comorbidity Index; ED, Emergency Department; MTS, Manchester Triage System; LOS, Length of Stay.
