# Supplementary material for: Appropriateness, Reasons and Independent Predictors of Consultations in the Emergency Department (ED) of a Dutch Tertiary Care Center: A Prospective Cohort Study
Source: PLoS One. 2016 Feb 19;11(2):e0149079. doi: 10.1371/journal.pone.0149079 (PMC4760948; doi:10.1371/journal.pone.0149079)
Supplement: S3 Table — (DOCX) [file pone.0149079.s004.docx]

**Supporting Information**

**Supporting Table 3. Patient characteristics of patient with mandatory and none mandatory consultations.**

|  | | **Mandatory consultation** | **None mandatory consultation** |
| --- | --- | --- | --- |
| **N** (%) | | 169 | 155 |
| **Demographics** | |  |  |
|  | Age, mean (SD) | 54 (28) | 56 (22) |
|  | Sex (male, %) | 89 (53) | 84 (54) |
|  | Pediatrics (%) | 29 (17) | 11 (7) |
| **CCI**, mean (SD) * | | 1.21 (2.00) | 1.41 (1.84) |
|  | Low (%) | 148 (88) | 133 (86) |
|  | High (%) | 21 (12) | 22 (14) |
| **Referral status** | |  |  |
|  | Self-referral | 74 (44) | 63 (41) |
|  | General practitioner | 58 (34) | 69 (45) |
|  | Specialist | 37 (22) | 23 (15) |
| **Arrival by ambulance** | | 94 (56) | 74 (48) |
| **Triage category** ** | |  |  |
|  | Red | 4 (2) | 1 (1) |
|  | Orange | 57 (34) | 52 (34) |
|  | Yellow | 87 (51) | 83 (54) |
|  | Green | 20 (11) | 19 (12) |
|  | Blue | 1 (1) | 0 (0) |
| **Treating physician** | |  |  |
|  | ED physician | 123 (73) | 89 (57) |
|  | Other specialist | 46 (27) | 66 (43) |
| **Disposition** | |  |  |
|  | Home | 29 (17) | 43 (28) |
|  | Outpatient follow-up | 42 (25) | 46 (30) |
|  | Admission ward | 83 (49) | 65 (42) |
|  | Admission ICU | 15 (9) | 1 (1) |
|  | Other | 0 (0) | 0 (0) |
| **ED LOS** (minutes), median (IQR) [80] | | 193 (71 to 236) | 227 (156 to 301) |

Patient characteristics are presented for patients who received one or multiple obliged consultations and patients who received one or multiple none obliged consultations. Continuous data are presented as mean (SD) or median (IQR) and categorical data as frequency (%). Patients that receiving an obliged and none obliged consultation were considered in the obliged consultation cohort (N=16). The number of missing cases are noted between square brackets for each variable. Revisits ≤ 48 hours includes only patients that revisited the ED unanticipated with a complaint related to the index visit. * Patients with a CCI of ≤3 were classified as low and those with a CCI ≥4 as high. ^#^ The presented ‘triage category’ was according to the MTS. Abbreviations: CCI, Charlson Comorbidity Index; ED, Emergency Department; ICU, Intensive Care Unit; MTS, Manchester Triage System; LOS, Length of Stay.
