# Supplementary material for: Appropriateness, Reasons and Independent Predictors of Consultations in the Emergency Department (ED) of a Dutch Tertiary Care Center: A Prospective Cohort Study
Source: PLoS One. 2016 Feb 19;11(2):e0149079. doi: 10.1371/journal.pone.0149079 (PMC4760948; doi:10.1371/journal.pone.0149079)
Supplement: S4 Table — (DOCX) [file pone.0149079.s005.docx]

**Supporting Information**

|  | **Variance Inflation Factor** |
| --- | --- |
| **Age** | 1.172 |
| **CCI** | 1.075 |
| **Triage category** | 1.206 |
| **Treating physician** | 1.128 |
| **Arrival by ambulance** | 1.213 |
| **Triage Complaint** | 1.119 |

**Supporting Table 4. The Variance Inflation Factors of the independent predictors of consultation**

To assess collinearity the variance inflation factors were analysed. A factor below 3 indicated that there is no multicollinearity. All the variable used in the multivariable regression analysis of Table 5 had a variance inflation factor below 3 and therefore multicollinearity does not affect the odds ratios presented in Table 5. Abbreviations: CCI, Charlson comorbidity index.
